# Balance and Gait of Frail, Pre-Frail, and Robust Older Hispanics

**DOI:** 10.3390/geriatrics3030042

**Published:** 2018-07-18

**Authors:** Edgar R. Vieira, Rubens A. da Silva, Maria T. Severi, Alexandre C. Barbosa, Benjamin C. Amick III, Juan C. Zevallos, Iveris L. Martinez, Paulo H. M. Chaves

**Affiliations:** 1 Department of Physical Therapy, Florida International University, Miami, FL 33199, USA; ezaseveri@gmail.com; 2 Department of Health Sciences, University of Quebec at Chicoutimi, Chicoutimi, QC G7H 2B1, Canada; Rubens_DaSilva@uqac.ca; 3 Department of Physical Therapy, Federal University of Juiz de Fora, Governador Valadares, MG 35010-180, Brazil; alexwbarbosa@hotmail.com; 4 Department of Health Policy & Management, Florida International University, Miami, FL 33199, USA; bamickii@fiu.edu; 5 Department of Medical and Health Sciences Research, Florida International University, Miami, FL 33199, USA; juzevall@fiu.edu (J.C.Z.); pdehendo@fiu.edu (P.H.M.C.); 6 College of Health and Human Services, California State University, Long Beach, CA 90815, USA; iverism@gmail.com

**Keywords:** older adults, frailty, gait, balance, function, mobility, Hispanics

## Abstract

Older Hispanics are an understudied minority group in the US, and further understanding of the association between frailty, gait and balance impairments in disadvantaged older Hispanics is needed. The objectives of this study were to compare the balance and gait of older Hispanics by their frailty status. Sixty-three older Hispanics (21 men, 42 women, mean age 75 ± 7 years) attending senior centers in disadvantaged neighborhoods were grouped by their frailty status and completed balance and walking tests at a preferred speed and during street crossing simulations. Sixteen percent (*n* = 10) of the participants were frail, 71% (*n* = 45) were pre-frail, and 13% (*n* = 8) were robust. Frail participants had poorer balance than robust participants (F = 3.5, *p* = 0.042). The preferred walking speed of frail and pre-frail participants was lower (F = 6.3, *p* < 0.011) and they took shorter steps (F > 3.5, *p* = 0.002) than robust participants. During street crossing conditions, frail participants had wider steps (F = 3.3, *p* = 0.040), while pre-frail participants walked slower (F = 3.6, *p* = 0.032), and both took shorter steps than robust participants (F > 3.5, *p* < 0.043). Frailty and pre-frailty were prevalent and associated with gait and balance impairments in disadvantaged older Hispanics. The findings can inform the development of programs and interventions targeting this vulnerable underserved population.

## 1. Introduction

According to the US Census Bureau report published in August 2017, 57.5 million Hispanics were living in the US as of 1 July 2016 [1]. The population of older Hispanics (age 65 years and older) in the US is projected to increase by 112% between 2016 and 2030 [2]. Financial and other stresses are related to frailty in older Hispanics [3]. Frailty is a clinical syndrome in which ≥3 of the following are present: unintentional weight loss ≥10 lbs in past year, self-reported exhaustion, grip weakness, slow gait and low physical activity level [4]. Frail and pre-frail older Hispanics have lower health-related quality of life [5]. Pre-frail and frail statuses predicted disability in activities of daily living in 10-year follow up of 1645 older Hispanics [6]. Frailty is characterized by decreased reserve, diminished strength, endurance, resistance to stressors, and reduced physiological function, resulting in a state of high vulnerability for dependency and death [7]. Older Hispanics were found to fall more often than older Caribbeans (40% vs. 24%, *p* = 0.022) [8]. Also, frailty status, pre-frailty, poor balance and prior falls increased the risk of falling among 847 older Hispanics [9]. Hispanics have different anthropometry and health behaviors related to nutrition and physical activity than non-Hispanic Whites [10]. Despite higher rates of poverty, lower educational levels and less access to health care, Hispanics have been found to have better health outcomes than non-Hispanic Whites (Hispanic health paradox) [11]. These unique characteristics may be related to different gait and balance characteristics at older age. These potential differences may be even more apparent when frailty is involved. Older Hispanics are an understudied minority group in the US, and further understanding of the association between frailty, gait and balance impairments in disadvantaged older Hispanics is needed. Therefore, the objective of this study was to assess if balance and gait characteristics vary by frailty status in older Hispanics in senior centers in disadvantaged neighborhoods. 

## 2. Materials and Methods

**Figure 1:**
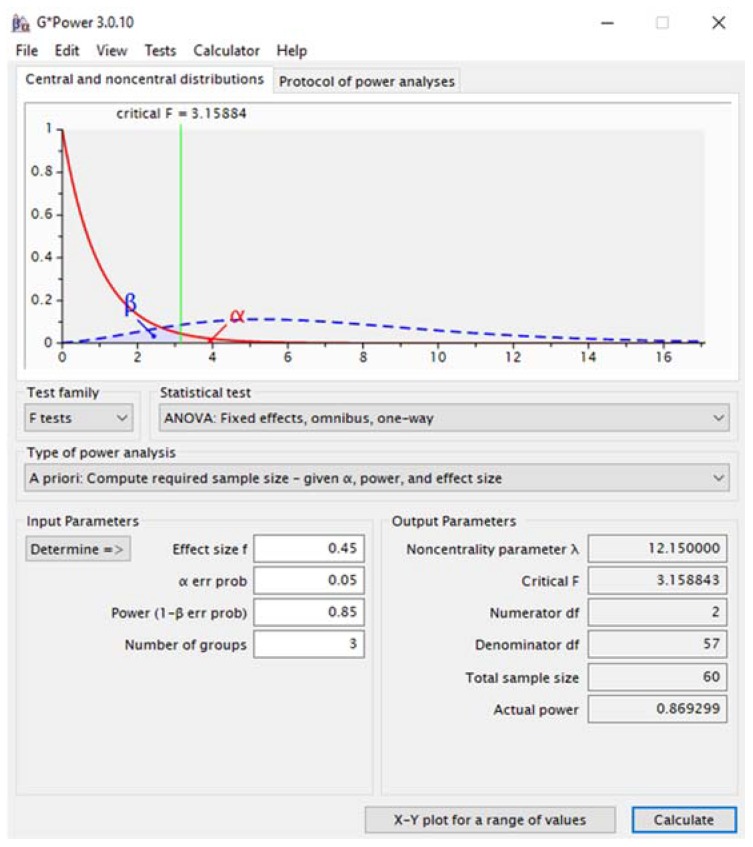
Sample size calculation.

Sixty-three older Hispanics (21 men, 42 women, mean age 75 ± 7 years) participated in this cross-sectional study at senior centers in disadvantaged neighborhoods in Miami-Dade, Florida. For sample size calculation, we used the G*Power program (v. 3.0.10, Franz Faul, University of Kiel, Germany). Considering an effect size of 0.45 to identify differences in gait temporospatial parameters with 0.05 α level [12], for three groups (frailty statuses) and two conditions (preferred walking versus walking during street crossing simulation) we would need a total sample size of 60 subjects for a 0.85 power. A screen shot of the inputs and calculation results is presented in Figure 1.

Participants were eligible for the study if they were Hispanic (self-reported), 65 years or older, and could walk 25 feet with or without an assistive device. This was the distance that they had to walk during the testing trials. That was to ensure that they could complete the assessment. The exclusion criteria were (1) an inability to provide informed consent (decisionally impaired), or to understand the study procedures or instructions (cognitive impairment); (2) a systolic blood pressure over 180 mmHg and/or diastolic blood pressure over 110 mmHg, and (3) headaches or visual blurring. 

All procedures performed in studies involving human participants were in accordance with the ethical standards of the institutional and/or national research committee and with the 1964 Helsinki declaration and its later amendments or comparable ethical standards. The study procedures and protocols were approved by the Institutional Review Board (IRB-14-0332). Before the assessment, participants signed an informed consent form and questions were addressed. 

The assessments lasted between 1 and 1.5 h per participant, and they were conducted using the participants’ preferred language (most often Spanish). Participants were told they were free to stop participating at any time. The participants received a report with the findings of their assessment and educational materials about the importance of exercising to maintain and recover physical function and prevent or treat frailty. The report included recommendations to discuss the findings with their primary care physician and/or physical therapist when needed. 

*Independent Variable: Frailty status.* Frailty was classified based on the following criteria: (1) unintentional weight loss of 10 lbs or more in the past year; (2) self-reported exhaustion 3 or more days per week; (3) grip strength <23 lbs for women and <32 lbs for men; (4) walking speed <80 cm per second, and (5) reporting to sit quietly and/or laying down for the vast majority of the day [13]. People who present none of the criteria are classified as robust, people who present one or two of the criteria are classified as pre-frail, and people who present three or more of them are classified as frail. 

*Dependent Variables: Balance and gait.* The participants’ balance was assessed using the Berg balance test [14]. The Berg balance test is a comprehensive assessment of static and dynamic balance in 14 tasks: sit-to-stand, standing unsupported, sitting unsupported, stand-to-sit, transfers, standing with eyes closed, standing with feet together, reaching forward with an outstretched arm, retrieving an object from the floor, turning the trunk with feet fixed, turning 360°, stool stepping, tandem standing, and standing on one limb [14]. For the gait assessment, the participants walked on an instrumented mat (GAITRite^®^, SN: Q209, CIR Systems Inc., Franklin, NJ, USA) at preferred speed (condition 1) and during street crossing simulations with regular (condition 2) and reduced (condition 3) crossing times [12]. During the simulated street crossing conditions, the participants were instructed to cross the walkway when the pedestrian light on the video indicated that it was safe to initiate crossing and to complete crossing before the countdown ended. A projector (BenQ^®^ MS504 SVGA DLP, Taipei, Taiwan) displayed a video of an intersection onto a screen placed 2 m beyond the end of the walkway. The video included traffic noise and displayed an intersection situation with cars crossing. The subjects were positioned on the sidewalk at the intersection looking at oncoming traffic from both directions; a pedestrian light was also displayed on the screen. We used street crossing simulations because it is a functional dual-task commonly performed during the activities of daily living that simultaneously challenge motor and cognitive functions [12]. For all conditions, the participants initiated their gait 2 m before the beginning of the mat and stopped two meters after the end of the mat, by the projection screen. Therefore, we evaluated steady state walking by removing the acceleration and deceleration phases. The participants completed one familiarization walking trial followed by three testing trials under each condition in randomized order. 

The following gait parameters were assessed: velocity—walking speed in cm/s calculated as distance covered divided by the ambulation time; cadence—number of steps per minute; step length—distance in cm between the heel center of 1 foot to the heel center of other foot during heel strike; step width—the distances in cm between a line linking the center of 1 foot during 2 subsequent steps and the center of the opposite foot during mid stance; swing and stance time—time from toe off to heel strike and time from heel strike to toe off; and single and double support time—time that one or both feet are on the floor simultaneously.

For the data analysis, potential differences in the proportions of males and females between groups were assessed using Chi-square (χ^2^). The homogeneity of the data was checked using Levene’s test. Data distribution was evaluated using the Shapiro–Wilk’s Test. All variables were normally distributed. Independent samples t-tests were used to compare the participants’ age and body mass index. The gait parameters were averaged across three trials under each condition. All statistical analyses were performed using the statistical package for the social sciences (SPSS version 20.0 for Windows, IBM, Chicago, IL, USA). Differences among groups on gait and balance parameters were tested using independent samples ANOVA and Tukey’s post-hoc tests within gait condition for each dependent variable. The statistical significant level cut-off point was set to an alpha level of 0.05. 

## 3. Results

From the 63 older Hispanics, 16% (*n* = 10) were frail, 71% (*n* = 45) were pre-frail, and 13% (*n* = 8) were robust. Approximately 70% of the frail and pre-frail participants were women, while two out of three robust participants were men (Table 1). 

There were no significant differences in age and body mass index among groups, but frail older Hispanics had poorer balance than the robust ones (*p* = 0.042). There were significant differences in gait velocity, step length and step width among the groups. On the other hand, there were no significant differences in cadence, swing and stance time, or single and double support time among the groups. Table 2 presents the comparisons among the frailty groups during the walking conditions for the variables that presented significant differences. The older Hispanics who were frail or pre-frail walked slower with shorter steps than those who were robust at preferred speed and during street crossing with regular time. During the latter, the robust had a smaller step width than the frail group (*p* = 0.040), and during the street crossing with reduced time, the frail participants took shorter steps than the ones that were robust (*p* = 0.041).

## 4. Discussion

We found a high prevalence of frailty and pre-frailty in disadvantaged older Hispanics, and the participants with this clinical syndrome presented gait and balance impairments. Frailty (16%) and pre-frailty (71%) were prevalent in our sample. In contrast, a study evaluating 1645 older Hispanics found that only 4% were frail and 46% were pre-frail [6]. In another study including 1996 older Hispanics, the proportions were 8% frail and 47% pre-frail [15]. Therefore, 50% to 55% were frail or pre-frail compared to 87% in our sample. The differences may be related to the fact that they studied frailty among older Mexican Americans in Texas, New Mexico, Colorado, Arizona and California, while most of our sample of older Hispanics in South Florida was from the Caribbean or South America (e.g., Cuban Americans, Porto Ricans, and Venezuelans). In addition to the differences in descent, the previous studies included subjects from different socio-economic status, while we assessed older Hispanics in senior centers located in impoverished neighborhoods. Frailty status is associated with the residence neighborhood’s ethnic composition and economic environment [16]. People living in impoverished neighborhoods often have inadequate public transportation, making exercise programs at senior centers inaccessible for most, even when such programs are available for a modest or no charge. In addition, the sample of our study was small; much smaller than the samples of the other studies mentioned. Therefore, our data on the prevalence of frailty and pre-frailty is not epidemiological in nature and should not be generalized to the overall population of older Hispanics. Selection bias may explain the higher rate of frailty and pre-frailty in our sample, and the findings should be interpreted with caution. 

Approximately 70% of the participants who were frail or pre-frail were women, while two out of the three robust participants were men. Frailty was previously found to be associated with female sex in Older Hispanics [17]. Frailty in older Hispanics was also associated with impairments in balance and gait; those who were frail had poorer balance than those who were robust. The scientific contributions of this study include the fact that, even though the sample is small and larger studies are necessary, the results indicate that the prevalence of frailty and pre-frailty may be high among socioeconomically disadvantaged older Hispanics in South Florida (e.g., Cuban Americans, Porto Ricans, and Venezuelans). In addition, the study quantified the impairments in different gait parameters (e.g., velocity, step length and width) by frailty status, which may be related with falls in this population. The gait and balance impairments among frail older Hispanics may help to explain the previously observed high rates of falls in this group; the odds of falling have been found to be higher in Hispanics that are frail or pre-frail, have poor balance, and/or had prior falls [9]. Falls are the number one cause of injury, hospitalization, and injury-related disability in older adults [18]. Falls in older adults can result from gait impairments, declines in balance, lower limb muscle strength and range of motion [19]. Considering the high prevalence of frailty, balance and gait impairments encountered in disadvantaged older Hispanics, prevention and treatment programs targeting this vulnerable population are needed and could be offered at senior centers. 

The limitations of this study include the fact that our study was cross-sectional, and therefore we do not know if frailty caused or is a consequence of the balance and gait impairments. These health issues are interconnected and, therefore, most likely each one can affect and contribute to the other. Longitudinal studies are required to evaluate the different pathways of frailty, gait and balance impairment development and progression. Another limitation is that our sample was small and larger studies of disadvantaged older Hispanics are required. Also, recall bias and misreporting is always a concern when using self-reported measures (e.g., weight loss, felling exhaustion, sitting or lying down frequency). This is a limitation of the frailty classification system used and may have resulted is some misclassifications. The lack of information on comorbidities and covariate adjustments is also a limitation of the study. In addition, we did not find studies comparing gait and balance by frailty status using similar methods we used. Therefore, we based our expected differences for the sample size calculation on a study that used similar methods to compare younger and older adults [12]. The study may have limited transferability. However, the actual (post-hoc) effect size for the comparison between the preferred gait speed (main variable) of frail vs. robust participants was 0.50, which is similar to the 0.45 used in the calculation. Despite this, larger studies are necessary. 

## 5. Conclusions

Frailty and pre-frailty were prevalent and associated with gait and balance impairments in disadvantaged older Hispanics. However, this conclusion needs to be considered with caution because the sample size was small; larger studies are needed. Directed prevention and treatment programs need to be offered at senior centers hosting older Hispanics.

## Figures and Tables

**Table 1:** Participants’ characteristics by frailty status.

Characteristics	1 Frail	2 Pre-Frail	3 Robust	ANOVA F, *p*	Post-Hoc Direction
Females, % (n)	70 (7)	73 (33)	25 (3)	3.9, *0.026* *	3 < 1 and 2
Age in years, mean (SD)	76 (7)	75 (7)	71 (5)	1.9, *0.157*	--
Body mass index in kg/m^2^, mean (SD)	29 (7)	30 (5)	27 (3)	1.2, *0.313*	--
Berg balance score, mean (SD)	50 (5)	51 (4)	55 (2)	3.5, *0.038* *	3 > 1

* Statistically significant difference among groups (*p* < 0.05).

**Table 2:** Gait parameters by frailty status (means ± standard deviations).

Parameters	Groups	Preferred Speed	Regular Time	Reduced Time
**Velocity (cm/s)**	1 Frail	100 ± 19	114 ± 23	119 ± 30
	2 Pre-Frail	102 ± 16	114 ± 18	121 ± 23
	3 Robust	125 ± 24	135 ± 24	138 ± 24
	ANOVA	F = 6.3, *p = 0.003* *	F = 3.6, *p = 0.035* *	F = 1.9, *p = 0.165*
	Post-hoc	3 > 1 and 2	3 > 1 and 2	--
**Step Length (cm)**	1 Frail	55 ± 8	59 ± 13	60 ± 13
	2 Pre-Frail	57 ± 7	61 ± 8	63 ± 9
	3 Robust	67 ± 9	70 ± 9	71 ± 9
	ANOVA	F = 7.4, *p = 0.001* *	F = 4, *p = 0.024* *	F = 3.6, *p = 0.032* *
	Post-hoc	3 > 1 and 2	3 > 1 and 2	3 > 1
**Step Width (cm)**	1 Frail	12 ± 2	12 ± 2	12 ± 3
	2 Pre-Frail	11 ± 3	11 ± 3	11 ± 3
	3 Robust	9 ± 3	8 ± 3	8 ± 3
	ANOVA	F = 2.5, *p = 0.091*	F = 3.3, *p = 0.042* *	F = 2.3, *p = 0.109*
	Post-hoc	--	3 < 1	--

* Statistically significant difference among groups (*p* < 0.05).
